# Association of time spent on social media with youth cigarette smoking and e-cigarette use in the UK: a national longitudinal study

**DOI:** 10.1136/thorax-2023-220569

**Published:** 2024-05-16

**Authors:** Nicholas S Hopkinson, Charlotte Vrinten, Jennie C Parnham, Márta K Radó, Filippos Filippidis, Eszter P Vamos, Anthony A Laverty

**Affiliations:** 1 National Heart and Lung Institute, Imperial College London, London, UK; 2 Department of Primary Care and Public Health, Imperial College London School of Public Health, London, UK; 3 Department of Medical Epidemiology and Biostatistics, Karolinska Institutet, Stockholm, Sweden

**Keywords:** Smoking, Tobacco control

## Abstract

**Background:**

Social media may influence children and young people’s health behaviour, including cigarette and e-cigarette use.

**Methods:**

We analysed data from participants aged 10–25 years in the UK Household Longitudinal Study 2015–2021. The amount of social media use reported on a normal weekday was related to current cigarette smoking and e-cigarette use. Generalised estimating equation (GEE) logistic regression models investigated associations of social media use with cigarette smoking and e-cigarette use. Models controlled for possible confounders including age, sex, country of UK, ethnicity, household income and use of cigarette/e-cigarettes by others within the home.

**Results:**

Among 10 808 participants with 27 962 observations, current cigarette smoking was reported by 8.6% of participants for at least one time point, and current e-cigarette use by 2.5% of participants. In adjusted GEE models, more frequent use of social media was associated with greater odds of current cigarette smoking. This was particularly apparent at higher levels of use (eg, adjusted odds ratio (AOR) 3.60, 95% CI 2.61 to 4.96 for ≥7 hours/day vs none). Associations were similar for e-cigarettes (AOR 2.73, 95% CI 1.40 to 5.29 for ≥7 hours/day social media use vs none). There was evidence of dose–response in associations between time spent on social media and both cigarette and e-cigarette use (both p<0.001). Analyses stratified by sex and household income found similar associations for cigarettes; however, for e-cigarettes associations were concentrated among males and those from higher household income groups.

**Conclusions:**

Social media use is associated with increased risk of cigarette smoking and e-cigarette use. There is a need for greater research on this issue as well as potential policy responses.

**WHAT IS ALREADY KNOWN ON THIS TOPIC**

- There is substantial use of social media among children and young people, which has had debated impacts on health outcomes. There are studies examining social media use and associations with cigarette and e-cigarette use in the US but only two such studies in the UK. One study was cross-sectional, while one previous cohort study of data from 2014 to 2018 found that social media use at age 14 years was associated with a greater likelihood of cigarette smoking at age 17 years. This study did not, however, assess the use of e-cigarettes.

**WHAT THIS STUDY ADDS**

- This study examined daily use of social media among 10–25-year-olds from 2015 to 2021. It found that time spent on social media is associated, in a dose-dependent manner, with likelihood both of cigarette smoking and vaping. Those using social media for ≥7 hours/day were more than two and a half times more likely to use both cigarettes and e-cigarettes than those not using social media.

**HOW THIS STUDY MIGHT AFFECT RESEARCH, PRACTICE OR POLICY**

- This study highlights that more frequent social media use is associated with increased likelihood of using both cigarettes and e-cigarettes in the UK. This reinforces concerns that social media is a vector of direct and indirect marketing and promotion of these products and that policies to curtail this may be warranted.

## Introduction

Understanding the mechanisms that drive uptake and use of cigarettes and e-cigarettes is key to developing strategies to prevent harm. The use of social media has been identified as a novel potential vector, with substantial increases in time spent in this activity by young people.^1–5^ Social media use increases with age, and girls are more likely to spend longer periods of time on social media than boys.^6^ Social media may be driving cigarette smoking and e-cigarette use through both direct, targeted advertisements and the use of paid influencers by the tobacco industry.^7^ To date, most evidence on the impact of social media on cigarette and e-cigarette use has focused on America.^8–10^ This has found associations with uptake, regular use and reduced perceptions of harm and has included assessment of engagement with different platforms.^11–13^ The only two previous UK studies include a cohort study which found that social media use at age 14 years was associated with greater likelihood of cigarette use at age 17 years.^14^ A second cross-national study from 42 countries including the UK concluded that there was a link between social media use and substance use but did not examine cigarette use separately from other substances.^15^

Previous research has identified links between social media use and both cigarette and e-cigarette use. For example, analyses of Instagram have identified networks of influencers promoting e-cigarettes, often without disclosing financial relationships; while Juul has recently settled a lawsuit over marketing of e-cigarettes to teens, including on social media.^16 17^ Comparative analyses in the UK have found good compliance with advertising standards for e-cigarettes on traditional media, but high levels of breaches on social media.^18^ Analyses of 11 of the most popular social media platforms have highlighted high levels of tobacco promotion, with few platforms having policies to deal with novel forms of promotion such as sponsored or influencer content.^19^ A systematic review of exposure to tobacco promotion and use identified 29 studies (none from the UK) and concluded that there is a need for greater regulation.^20^ Any proposal to regulate social media needs to be justified and based on evidence. To contribute to this, we examined the longitudinal relationship of social media use with cigarette smoking and e-cigarette use among children and young people in the UK.

## Methods

Data come from participants of the UK Household Longitudinal Study (UKHLS), also known as Understanding Society.^21^ This is a longitudinal household panel study with annual surveys starting in 2009. The original sample consisted of a clustered and stratified probability sample of approximately 28 000 households in the UK. Data are collected via face-to-face interviews carried out by a trained interviewer in the respondent’s home and via online, self-completion questionnaires. Adults over the age of 16 years or above are asked to complete an individual questionnaire, including a self-completion questionnaire. Household members aged 10–15 years are asked to fill in a shorter self-completion questionnaire, with permission from their parent or carer.

We have focused on children and young adults aged 10–25 years using data from 2015/2016 to 2020/2021 (wave 7 to wave 12). Questions on e-cigarette use were added to UKHLS in 2015/2016. Participation in the panel is voluntary, with a gift voucher sent to encourage completion of questionnaires and a further gift voucher sent when these are completed. All participants provided consent to be interviewed. The University of Essex Ethics Committee approved all data collection.^22^

### Outcomes and exposure

We used three separate binary outcomes: current cigarette smoking, current e-cigarette use and current dual use of both products. Participants were classified as current cigarette smokers if they responded “I usually smoke between one and six cigarettes a week” or “I usually smoke more than six cigarettes a week”. All other responses were coded as non-users. The same question was used for all waves of data and for all ages.

Current e-cigarette use was first assessed in 2015/2016 with the question “Do you ever use electronic cigarettes (e-cigarettes)?” with response options “Yes” and “No”. From wave 8 (2016/2017) onwards participants were classified as current (weekly) e-cigarette users if they responded “I use e-cigarettes at least once a week”. All other responses were coded as non-users. Dual use was classified as participants currently using both products, with those using only one or no products classed as non-dual users.

The main exposure variable was social media use. Participants were first asked “Do you belong to any social networking websites?” (Yes/No), and if “Yes”, they were also asked how many hours they spend chatting or interacting with friends through a social website on a normal weekday, with the following response options: “None”, “<1 hour/day”, “1–3 hours”, “4–6 hours” and “≥7 hours”. We combined those reporting “None” along with those who were not a member of a social media website into a reference category of “Not a member or no use”.^6^

### Covariates

We considered a range of potentially relevant sociodemographics: age, sex, country in UK, self-defined ethnic group (collapsed into White vs non-White due to low numbers in the non-White category), an indicator of living in an urban or rural areas (derived from Office for National Statistics Rural and Urban Classification of Output Areas) and equivalised household net income (based on the Organisation for Economic Co-operation and Development (OECD) equivalence scale, which was used to adjust household income by household composition^23^).

### Statistical analyses

We compared differences in sociodemographics between categories of social media use using ANOVA. We used binary generalised estimating equation (GEE) regression models (family: binomial; link: logit; correlation matrix: exchangeable) to assess relationships between social media use and product use, using separate models for each outcome: cigarette smoking, e-cigarette use and dual use. GEE models assess changes over time and account for the correlation caused by observations being from the same individuals.^24^ We also present tests for trend based on frequency of social media use. Analyses were adjusted for time (categorical) as well as the sociodemographic variables listed above. Models of cigarette smoking were additionally adjusted for parental tobacco use, models of e-cigarette use were adjusted for parental e-cigarette use, and models of dual use were adjusted for both. Analyses used survey weights designed by the UKHLS survey team to account for clustered and stratified probability sampling and non-response bias.^25^

We tested for interactions of social media use with age (split into above and below 18 years of age), sex and household income (in three groups). This was due to possible differences between those above and below the legal age of sale, greater social media use among women, and potentially differential effects by socioeconomic groups. All interactions were p<0.001 and so we present stratified analyses. Due to the small numbers, we did not test interactions for dual use.

### Sensitivity analyses

We performed a range of sensitivity analyses to test the robustness of our findings. As it is possible that those not using social media at all are atypical, we repeated our analyses excluding these participants. Our main analyses used household income as a marker of socioeconomic status. We also performed our analyses using Index of Multiple Deprivation (IMD) (in five groups) as an alternative marker of socioeconomic status. We performed analyses categorising current e-cigarette use as participants who reported using e-cigarettes at least monthly. We also performed analyses controlling for a measure of mental health (the 12-item General Health Questionnaire (GHQ-12)) to consider whether this is a possible pathway, whereby social media impacts mental health, which is then linked to cigarette and e-cigarette use.

Finally, we used fixed effects analyses to directly test if changes in social media use corresponded to uptake of cigarette smoking and e-cigarette use. These adjusted for the time-varying variables parental cigarette/e-cigarette use and household income. These models were on a smaller subset of individuals who were not product users when entering the study and who were found to change their social media use over time.

## Results

Outcomes and covariates across categories of social media use are shown in table 1. Overall, 8.6% of the sample reported current cigarette smoking at one or more data point, 2.5% reported current e-cigarette use, and 1.1% of participants were dual users at one or more data point. Social media use frequency broken down by covariates is shown in online supplemental appendix table 1.

10.1136/thorax-2023-220569.supp1 Supplementary data

**Table 1:** Description of sample observations by social media use 2015–2021

Covariate	Weekday social media use						P value for difference between groups
	None or not a member	<1 hour	1–3 hours	4–6 hours	≥7 hours	Total	
Current cigarette smoking (%)	2.0	6.3	9.2	12.2	15.7	8.6	<0.001
Current e-cigarette use (%)	0.8	2.0	2.4	3.8	4.0	2.5	<0.001
Dual use (%)	0.3	0.9	1.1	1.7	2.0	1.1	<0.001
Male (%)	57.5	52.4	45.3	38.3	39.9	47.1	<0.001
Mean age (years) (SD)	12.0 (2.7)	15.22 (4.0)	16.49 (3.6)	16.95 (3.0)	17.7 (2.6)	15.74 (3.8)	<0.001
White ethnicity (%)	65.6	71.4	75.1	75.0	74.2	72.8	<0.001
Urban areas (%)	79.4	78.1	77.4	79.6	81.4	78.5	<0.001
Mean household income (£) (SD)	1679 (3485)	1709 (1290)	1689 (1055)	1622 (1066)	1551 (960)	1670 (1666)	<0.001
Parental cigarette smoking (%)	17.0	16.3	18.6	21.0	25.2	18.7	<0.001
Parental e-cigarette use (%)	7.3	7.5	8.3	9.4	10.5	8.3	<0.001
Overall N	4356	7808	11 739	5110	2715	31 728	

Results are collapsed across all survey years.

**Figure 1:**
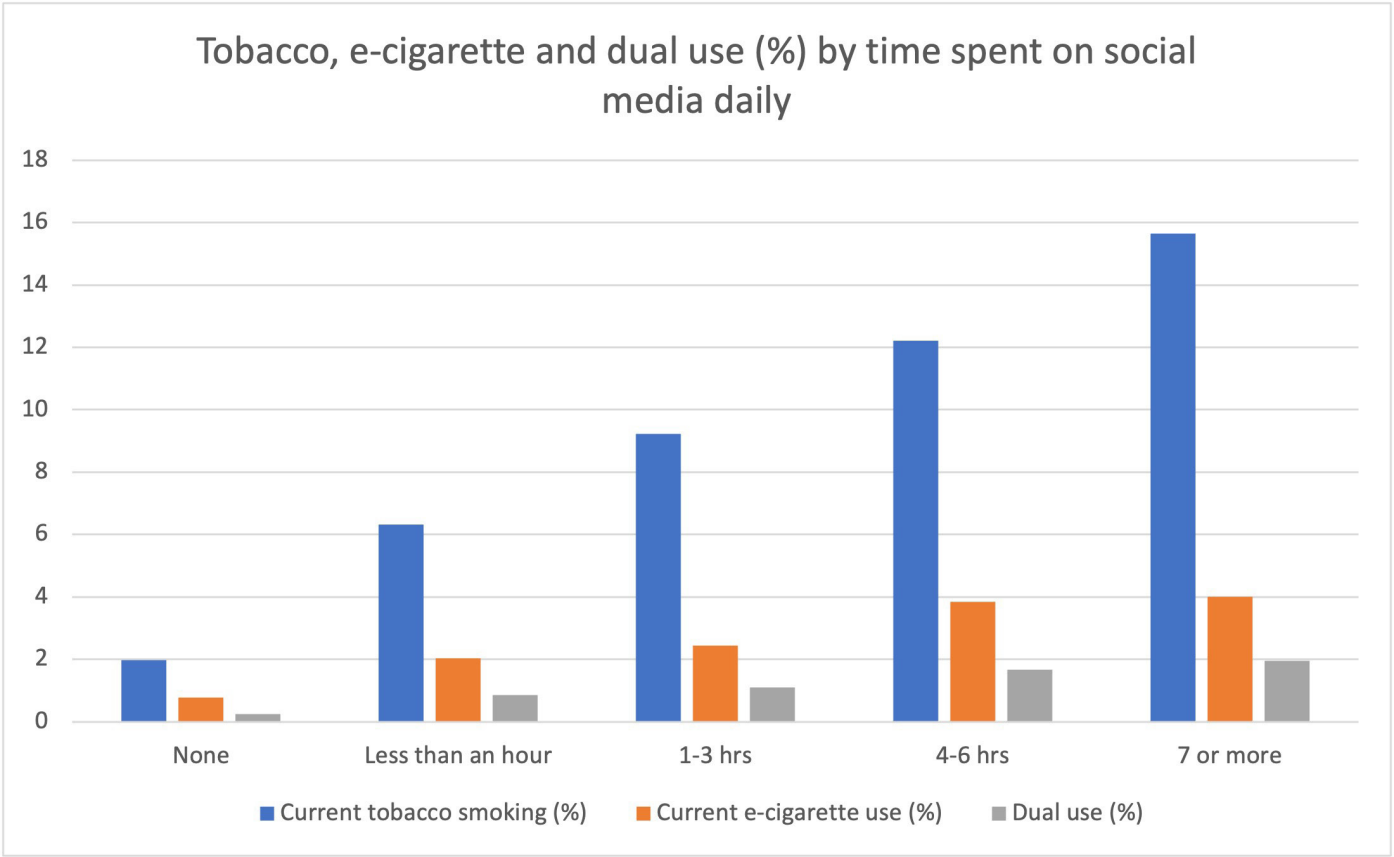
Cigarette smoking, e-cigarette use and dual use by social media use.

Cigarette smoking, e-cigarette use and dual use were all more common among participants reporting greater social media use (all p<0.001) (figure 1). Some 2.0% of participants who used social media “None or not a member” reported being a current cigarette smoker compared with 15.7% among those using social media for ≥7 hours/day. Current e-cigarette use ranged from 0.8% among those not using social media to 2.5% among those using it for ≥7 hours/day.

Differences between categories of social media use were apparent for all variables studied (all p<0.001). Males were less likely to be in higher social media use groups than females (57.5% of the “None or not a member” social media group compared with 39.9% of the “≥7 hours/day” group). Social media use was more frequent at older ages (mean age of “None or not a member” social media group 12.0 years vs 17.7 years for the “≥7 hours/day” group). Parental cigarette smoking was more common among those using social media more frequently (17.0% for the “None or not a member” social media group vs 25.2% for the “≥7 hours/day” group) as was parental e-cigarette use (7.3% and 10.5%, respectively).

Table 2 shows results of our GEE models of social media use and cigarette smoking. Cigarette smoking was more common among those using social media more frequently (p for trend <0.001). Those using social media for “<1 hour/day” were more likely to be current cigarette smokers than those using social media “None or not a member” (adjusted odds ratio (AOR) 1.92, 95% CI 1.43 to 2.58) (table 2). Those using social media for “≥7 hours/day” were substantially more likely to be current cigarette smokers than those using social media “None or not a member” (AOR 3.60, 95% CI 2.61 to 4.96).

**Table 2:** Associations of social media use with current cigarette use from generalised estimating equation model

Weekday social media use (hours/day)	AOR	P value	Lower CI	Upper CI
None or not a member	Ref.	Ref.	Ref.	Ref.
<1 hour	1.92	<0.001	1.43	2.58
1–3 hours	2.56	<0.001	1.93	3.41
4–6 hours	2.87	<0.001	2.12	3.87
≥7 hours	3.60	<0.001	2.61	4.96
Year (2015/2016)	Ref.	Ref.	Ref.	Ref.
2016/2017	1.12	0.132	0.97	1.29
2017/2018	0.99	0.896	0.85	1.16
2018/2019	1.38	<0.001	1.17	1.62
2019/2020	1.24	0.019	1.04	1.48
2020/2021	1.01	0.925	0.82	1.25
Sex (male vs female)	1.15	0.084	0.98	1.34
Age (years)	1.20	<0.001	1.18	1.23
England	Ref.	Ref.	Ref.	Ref.
Wales	0.77	0.115	0.55	1.07
Scotland	0.86	0.290	0.65	1.14
Northern Ireland	1.07	0.608	0.82	1.41
White ethnicity	Ref.	Ref.	Ref.	Ref.
Non-White ethnicity	0.57	<0.001	0.43	0.76
Rural area vs urban	1.03	0.782	0.85	1.23
Lowest household income group	Ref.	Ref.	Ref.	Ref.
Middle household income group	0.84	0.011	0.74	0.96
Highest household income group	0.74	<0.001	0.63	0.86
Parental smoking yes vs no	2.91	<0.001	2.49	3.40

AOR, adjusted odds ratio; CI, confidence interval; Ref., reference.

Table 3 shows results for e-cigarette use. E-cigarette use was more common among those using social media more frequently (p for trend <0.001). E-cigarette use was more common among those using social media “1–3 hours per day” compared with those using it “None or not a member” (AOR 1.92, 95% CI 1.07 to 3.46). E-cigarette use was considerably more likely among participants using social media “≥7 hours/day” than those using social media “None or not a member” (AOR 2.73, CI 1.40 to 5.29).

**Table 3:** Associations of social media use and current e-cigarette use from generalised estimating equation model

Weekday social media use (hours/day)	AOR	P value	Lower CI	Upper CI
None or not a member	Ref.	Ref.	Ref.	Ref.
<1 hour	1.42	0.231	0.80	2.54
1–3 hours	1.92	0.030	1.07	3.46
4–6 hours	3.10	0.000	1.70	5.63
≥7 hours	2.73	0.003	1.40	5.29
Year (2015/2016)	Ref.	Ref.	Ref.	Ref.
2016/2017	0.21	<0.001	0.15	0.30
2017/2018	0.31	<0.001	0.24	0.41
2018/2019	0.41	<0.001	0.31	0.54
2019/2020	0.38	<0.001	0.27	0.54
2020/2021	0.43	<0.001	0.30	0.62
Sex (male vs female)	2.33	<0.001	1.81	3.00
Age (continuous)	1.20	<0.001	1.17	1.24
England	Ref.	Ref.	Ref.	Ref.
Wales	0.71	0.192	0.42	1.19
Scotland	0.73	0.130	0.48	1.10
Northern Ireland	0.82	0.398	0.53	1.29
White ethnicity	Ref.	Ref.	Ref.	Ref.
Non-White ethnicity	0.65	0.030	0.44	0.96
Rural area vs urban	1.08	0.582	0.82	1.43
Household income group lowest	Ref.	Ref.	Ref.	Ref.
Household income group middle	0.89	0.383	0.68	1.16
Household income group highest	0.78	0.085	0.59	1.03
Parental e-cigarette use yes vs no	2.97	<0.001	2.24	3.93

AOR, adjusted odds ratio; CI, confidence interval; Ref., reference.

Table 4 shows results for dual cigarette and e-cigarette use. Models have wide confidence intervals reflecting low levels of dual use. Those using social media more frequently were more likely to be dual users (p for trend <0.001). Those using social media “1–3 hours per day” were more likely to be dual users compared with those using it “None or not a member” (AOR 3.28, 95% CI 1.24 to 8.70). Dual use was more likely among participants using social media “≥7 hours/day” than among those using social media “None or not a member” (AOR 4.96, 95% CI 1.71 to 14.34).

**Table 4:** Associations of social media use with current e-cigarette and cigarette dual use from generalised estimating equation model

Weekday social media use (hours/day)	AOR	P value	Lower CI	Upper CI
None or not a member	Ref.	Ref.	Ref.	Ref.
<1 hour	2.33	0.095	0.86	6.27
1–3 hours	3.28	0.017	1.24	8.70
4–6 hours	4.26	0.005	1.55	11.72
≥7 hours	4.96	0.003	1.71	14.34
Year (2015/2016)	Ref.	Ref.	Ref.	Ref.
2016/2017	0.19	<0.001	0.11	0.31
2017/2018	0.26	<0.001	0.17	0.40
2018/2019	0.42	<0.001	0.28	0.63
2019/2020	0.30	<0.001	0.18	0.51
2020/2021	0.32	<0.001	0.17	0.59
Sex (male vs female)	2.16	<0.001	1.54	3.02
Age (continuous)	1.19	<0.001	1.13	1.25
England	Ref.	Ref.	Ref.	Ref.
Wales	0.96	0.903	0.52	1.77
Scotland	0.73	0.255	0.43	1.25
Northern Ireland	0.95	0.854	0.53	1.68
White ethnicity	Ref.	Ref.	Ref.	Ref.
Non-White ethnicity	0.72	0.282	0.39	1.32
Rural area vs urban	1.21	0.304	0.84	1.75
Household income group lowest	Ref.	Ref.	Ref.	Ref.
Household income group middle	0.89	0.526	0.63	1.27
Household income group highest	0.68	0.060	0.46	1.02
Parental smoking yes vs no	2.10	0.000	1.47	3.02
Parental e-cigarette use yes vs no	1.94	<0.001	1.29	2.91

AOR, adjusted odds ratio; CI, confidence interval; Ref., reference.

Interactions of social media and sex were statistically significant for both cigarettes and e-cigarettes (both p<0.001). In stratified models (table 5) AORs were similar between the sexes for current cigarette smoking. For e-cigarettes, associations between social media use and e-cigarette use were statistically significant for males but not for females (AOR 4.10, 95% CI 1.90 to 8.87 for males for “≥7 hours/day” vs “None or not a member” social media use).

**Table 5:** Associations of social media use with current e-cigarette and cigarette use from gender stratified generalised estimating equation models

Weekday social media use (hours/day)	Males				Females			
	AOR	P value	Lower CI	Upper CI	AOR	P value	Lower CI	Upper CI
**Current cigarette use**								
None or not a member	Ref.	Ref.	Ref.	Ref.	Ref.	Ref.	Ref.	Ref.
<1 hour	1.83	0.003	1.23	2.74	2.01	0.002	1.30	3.10
1–3 hours	2.52	<0.001	1.70	3.75	2.59	<0.001	1.71	3.91
4–6 hours	2.81	<0.001	1.85	4.27	2.92	<0.001	1.90	4.49
≥7 hours	3.47	<0.001	2.22	5.44	3.72	<0.001	2.35	5.90
**Current e-cigarette use**								
None or not a member	Ref.	Ref.	Ref.	Ref.	Ref.	Ref.	Ref.	Ref.
<1 hour	1.85	0.068	0.95	3.59	0.89	0.811	0.34	2.35
1–3 hours	2.80	0.002	1.47	5.32	0.98	0.966	0.35	2.74
4–6 hours	4.97	<0.001	2.56	9.62	1.34	0.576	0.48	3.71
≥7 hours	4.10	<0.001	1.90	8.87	1.34	0.603	0.45	4.01

Results from models controlled for year, age, sex, country in UK, self-defined ethnic group (White vs non-White), an indicator of living in an urban or rural area, and equivalised household net income. Cigarette use models were adjusted for cigarette smoking by caregivers and e-cigarette models by use of e-cigarettes by caregivers.

AOR, adjusted odds ratio; CI, confidence interval; Ref., reference.

Interactions with household income categories were statistically significant (p<0.001 for both cigarettes and e-cigarettes) (table 6). In stratified analyses of cigarette smoking, point estimates for the richest income group were higher than for the lowest income group, although these overlapped (eg, AOR 5.22 for “≥7 hours/day”, 95% CI 2.82 to 9.67 for the richest income group vs AOR 4.17, 95% CI 2.27 to 7.65 for the lowest income group). For e-cigarette use, associations were statistically significant for the highest income groups (eg, AOR 7.85, 95% CI 1.72 to 35.82 for “≥7 hours/day” vs no social media use) but were not statistically significant for the lowest income group.

**Table 6:** Associations of social media use with current e-cigarette and cigarette use from household income stratified generalised estimating equation models

Weekday social media use (hours/day)	Lowest income group				Middle income group			
	AOR	P value	Lower CI	Upper CI	AOR	P value	Lower CI	Upper CI
**Current cigarette use**								
None or not a member	Ref.	Ref.	Ref.	Ref.	Ref.	Ref.	Ref.	Ref.
<1 hour	2.17	0.008	1.22	3.86	1.52	0.107	0.91	2.55
1–3 hours	3.06	<0.001	1.74	5.36	2.28	0.001	1.39	3.74
4–6 hours	3.69	<0.001	2.08	6.52	2.47	0.001	1.47	4.14
≥7 hours	4.17	<0.001	2.27	7.65	3	<0.001	1.74	5.17
–	–	–	–	–	Richest income group			
–	–	–	–	–	Ref.	Ref.	Ref.	Ref.
–	–	–	–	–	2.67	0.001	1.53	4.65
–	–	–	–	–	2.99	<0.001	1.73	5.18
–	–	–	–	–	3.41	<0.001	1.9	6.13
–	–	–	–	–	5.22	<0.001	2.82	9.67
**Current e-cigarette use**								
					AOR	P value	Lower CI	Upper CI
None or not a member	Ref.	Ref.	Ref.	Ref.	Ref.	Ref.	Ref.	Ref.
<1 hour	0.75	0.501	0.32	1.74	2.04	0.145	0.78	5.34
1–3 hours	0.87	0.735	0.39	1.94	2.61	0.069	0.93	7.34
4–6 hours	1.78	0.17	0.78	4.06	4.5	0.004	1.61	12.6
≥7 hours	1.65	0.301	0.64	4.23	4.51	0.008	1.49	13.69
–	–	–	–	–	Richest income group			
–	–	–	–	–	Ref.	Ref.	Ref.	Ref.
–	–	–	–	–	5.42	0.019	1.32	22.34
–	–	–	–	–	8.03	0.004	1.95	33.02
–	–	–	–	–	10.65	0.001	2.53	44.79
–	–	–	–	–	7.85	0.008	1.72	35.82

Results from models controlled for year, age, sex, country in UK, self-defined ethnic group (White vs non-White), an indicator of living in an urban or rural area, and equivalised household net income. Cigarette use models were adjusted for cigarette smoking by caregivers and e-cigarette models by use of e-cigarettes by caregivers.

AOR, adjusted odds ratio; CI, confidence interval; Ref., reference.

Analyses stratified by age found similar results to main analyses for cigarettes (online supplemental appendix table 2). Models for e-cigarette use were only statistically significant among those <18 years old.

### Sensitivity analyses

GEE analyses excluding those not using any social media were similar to main analyses (online supplemental appendix table 3). Analyses using IMD as a marker of socioeconomic status rather than household income also gave similar results (online supplemental appendix table 4). Analyses classifying current e-cigarette use as participants using them at least monthly also gave similar results although with larger point estimates (online supplemental appendix table 5). Analyses controlling for GHQ-12 as a measure of mental health were similar for cigarettes but did not find statistically significant associations between social media and e-cigarette or dual use. This may indicate that social media use impacts mental health, which in turn impacts likelihood of using cigarettes or e-cigarettes, although this result should be treated with caution (online supplemental appendix table 6).

Fixed effect analyses gave similar results to main analyses for uptake of cigarette smoking (online supplemental appendix table 7). It should be noted that sample size was much reduced for this model (n=864). These analyses found some evidence that changes in social media use are linked to uptake of cigarette smoking in a dose–response manner (p for trend=0.053). For example, changing to using social media for ≥7 hours/day was associated with more than double the odds of taking up cigarette smoking (AOR 2.33, 95% CI 1.28 to 4.24).

Associations between changes in social media use and uptake of e-cigarettes did not reveal associations between changes in social media use and uptake of e-cigarettes. These analysis models had even lower sample sizes (n=564). For example, AORs of e-cigarette uptake ranged from 0.71 (95% CI 0.34 to 1.48) for participants using social media “<1 hour/day” to AOR 0.84 (95% CI 0.38 to 1.85) for those using social media “≥7 hours/day”. The test for trend was not statistically significant (p=0.584).

## Discussion

The main finding of the present study is that in children and young adults more frequent social media use was associated with a higher likelihood of both current use of cigarettes and e-cigarettes. This association was independent of other factors associated with increasing smoking and vaping including age, gender, socioeconomic status and parental smoking and vaping. These findings were robust to sensitivity analyses, while in stratified analyses there were more consistent associations for e-cigarette use among those under the legal age of sale, males and those with higher household incomes.

While we were unable to assess use of specific social media platforms or what content was being accessed, we propose a number of possible, non-exclusive explanations for this relationship. First, and most straightforwardly, there is evidence that the corporations behind cigarette smoking and vaping make use of social media to advertise and promote their products.^8–10 16^ This includes direct advertising which is algorithmically targeted and the use of paid social media influencers who present smoking and vaping as a fashionable and desirable activity. Greater time spent on social media is likely to increase exposure to these forms of influence. While cigarettes and e-cigarettes are likely promoted differently, we found association with use of both products, highlighting the need for greater understanding of such corporate behaviours. Second, social media use has been shown to have features in common with reward-seeking addictive behaviour.^26^ High social media use may increase susceptibility to other addictive behaviours like smoking. Alternatively, both behaviours may be driven by a common susceptibility. Third, as a space that is largely unsupervised by parents/caregivers, social media use may encourage behaviours that are transgressive, including cigarette smoking and vaping. There is evidence that peer smoking is a strong influence on child uptake of smoking^27^ and social media is one of the ways in which peer smoking and vaping will be experienced, both by seeing others’ behaviour and by sharing “influencer content” that promotes these behaviours.

Stratified analyses revealed more consistent associations for cigarettes, while for e-cigarettes statistically significant associations were only found for those under the legal age of sale, among males, and those from richer households. Analyses of cigarette smoking did not identify changes over time, which fits with other evidence that smoking prevalence has been reasonably consistent over this time frame.^28^ Analyses of e-cigarette use found reduced odds of these outcomes after 2015/2016, likely caused by changes in e-cigarette use ascertainment, although our main findings were robust to reclassification to examine monthly use. Our main analyses focused on weekly use of e-cigarettes; as any health impacts are probably related to amounts of vapour inhaled, this measure of regular use is more important for health than e-cigarette experimentation.

### Strengths and limitations

This study uses a nationally representative cohort to examine social media use and use of cigarettes and e-cigarettes over time. UKHLS households are sampled based on geographical areas, population densities and ethnic composition, with survey weight adjusting for differential non-response across groups.^29^ We conducted a range of sensitivity analyses, although other potential factors such as education may also be important. All data are based on self-report, and specifically we do not have information about which social media platforms were being used or how individuals were using them, for example, the extent to which they are interacting socially with individuals they know or consuming content from influencers, personalities or media corporations, etc. Precise pathways remain to be fully elucidated: our sensitivity analyses point to a possible role for mental health, although it should be noted that a formal mediation analysis was outside the scope of this article. As cigarette smoking is linked to poorer mental health, these relationships could well be bidirectional.^30^ This, as well as potential targeted advertising, are among pathways that should be investigated in both quantitative and qualitative research.

### Policy implications

Although we do not have data on the specific platforms used or content used, there is compelling evidence that vape companies are using social media to market their products.^2–5^ The content that social media users are exposed to is to a substantial extent algorithmically controlled, both through targeted advertising and by the promotion of material that maximises engagement in order to increase revenue to the platform. This can be controlled. For example, far right imagery which is otherwise widely available is largely inaccessible in Germany, as a consequence of German law which social media platforms are bound to enforce. The companies that own social media platforms have substantial power to modify exposure to material that promotes smoking and vaping if they choose to or are compelled to. Voluntary codes seem unlikely to achieve this, and the introduction and enforcement on bans on material that promote this should be considered. In general, we think that algorithms should not be promoting products to individuals that they cannot legally buy. Legislation and enforcement around this and other corporate determinants of health concerns should be considered a core part of online safety and child protection.

## Conclusion

This longitudinal analysis of children and young people in the UK found that more frequent social media use is associated with an increased risk of cigarette and e-cigarette use.

## Data Availability

Data are available in a public, open access repository. Data available from UK Data Service https://ukdataservice.ac.uk.
