# Supplementary material for: Association of time spent on social media with youth cigarette smoking and e-cigarette use in the UK: a national longitudinal study
Source: Thorax. 2024 May 16;79(7):662–9. doi: 10.1136/thorax-2023-220569 (PMC11187353; doi:10.1136/thorax-2023-220569)
Supplement: Supplementary data [file thorax-2023-220569supp001.pdf]

APPENDIX

Associations of social media use with cigarette smoking and e-cigarettes: a national longitudinal study

Appendix Table 1: Social media use by socio-demographic variables

	Sex		Age group		Ethnicity		Area	
Daily social media use (hours/day)	Male	Female	Age < 18 years	Age 18 years +	White	Non White	Rural	Urban
None or not a member	11.0	16.7	19.9	2.1	12.3	17.3	13.9	13.2
Less than an hour	22.2	27.4	26.7	20.8	24.2	25.9	24.5	25.1
1-3 hrs	38.3	35.6	33.5	43.5	38.2	33.9	36.4	39.0
4-6 hrs	18.8	13.1	13.8	20.5	16.6	14.8	16.3	15.3
7 or more	9.7	7.3	6.1	13.1	8.7	8.1	8.9	7.4
Total	100	100	100	100	100	100	100	100
	Parental cigarette smoking		Parental e-cigarette use		Household income group			-
	No	Yes	No	Yes	Lowest	Middle	Highest	-
None or not a member	14.6	12.9	14.4	12.6	15.6	13.5	12.7	-
Less than an hour	25.3	21.3	24.8	22.1	24.0	24.5	25.4	-
1-3 hrs	36.8	36.4	36.7	36.5	34.6	36.9	39.5	-
4-6 hrs	15.6	18.0	15.8	18.2	16.4	16.6	15.1	-
7 or more	7.8	11.4	8.2	10.7	9.4	8.6	7.3	-
Total	100	100	100	100	100	100	100	-

Appendix Table 2: **Associations of social media use with product use from age stratified Generalised Estimating Equation models.**

Current cigarette smoking								
Daily social media use	Age 10-17 years				Age 18 - 25 years			
	AOR	p value	Lower CI	Upper CI	AOR	p value	Lower CI	Upper CI
None or not a member	ref	ref	ref	ref	ref	ref	ref	ref
Less than an hour	1.71	0.008	1.15	2.55	1.49	0.103	0.92	2.40
1-3 hours	2.73	<0.001	1.86	4.01	1.60	0.053	0.99	2.57
4-6 hours	3.35	<0.001	2.22	5.06	1.71	0.031	1.05	2.78
7 or more	4.52	<0.001	2.88	7.10	2.05	0.005	1.24	3.39
Current e-cigarette use								
None or not a member	ref	ref	ref	ref	ref	ref	ref	ref
Less than an hour	1.85	0.121	0.85	4.03	0.51	0.173	0.19	1.35
1-3 hours	2.47	0.018	1.17	5.25	0.60	0.308	0.23	1.59
4-6 hours	5.11	<0.001	2.38	10.99	0.76	0.578	0.28	2.03
7 or more	4.90	0.001	1.99	12.08	0.64	0.393	0.23	1.78

Results from models controlled for year, age, sex, country in UK, self-defined ethnic group (White vs. non-White), an indicator of living in an urban or rural areas, and equivalised household net income. Cigarette smoking models were adjusted for cigarette smoking by caregivers and e-cigarette models by use of e-cigarettes by caregivers.

Appendix Table 3: **Associations of social media use with product use from Generalised Estimating Equation models, excluding participants who were not a member or who used no social media**

Weekday social media use (hours)/day	AOR	p value	Lower CI	Upper CI
Current cigarette smoking				
Less than an hour	ref	ref	ref	ref
1-3 hrs	1.33	<0.001	1.15	1.53
4-6 hrs	1.47	<0.001	1.23	1.76
7 or more	1.86	<0.001	1.52	2.27
Current e-cigarette use				
Less than an hour	ref	ref	ref	ref
1-3 hrs	1.37	0.034	1.02	1.82
4-6 hrs	2.21	<0.001	1.60	3.05
7 or more	1.96	0.001	1.31	2.94
Current dual use (cigarette and e-cigarettes)				
Less than an hour	ref	ref	ref	ref
1-3 hrs	1.43	0.092	0.94	2.17
4-6 hrs	1.86	0.010	1.16	2.98
7 or more	2.19	0.005	1.27	3.79

AOR = adjusted odds ratio, CI = confidence interval

Results from models controlled for year, age, sex, country in UK, self-defined ethnic group (White vs. non-White), an indicator of living in an urban or rural areas, and equivalised household net income. Cigarette smoking models were adjusted for cigarette smoking by caregivers and e-cigarette models by use of e-cigarettes by caregivers. Dual use models were adjusted for use of both e-cigarettes and cigarettes by caregivers.

Appendix Table 4: **Associations of social media use with product use from Generalised Estimating Equation models, using IMD as a marker of socio-economic status**

Weekday social media use (hours/day)	AOR	p value	Lower CI	Upper CI
Current cigarette smoking				
None or not a member	ref	ref	ref	ref
Less than an hour	1.94	<0.001	1.45	2.61
1-3 hrs	2.62	<0.001	1.97	3.49
4-6 hrs	2.94	<0.001	2.18	3.96
7 or more	3.65	<0.001	2.65	5.02
Current e-cigarette use				
None or not a member	ref	ref	ref	ref
Less than an hour	1.45	0.202	0.82	2.58
1-3 hrs	1.95	0.026	1.08	3.50
4-6 hrs	3.26	<0.001	1.80	5.89
7 or more	2.71	0.003	1.40	5.24
Current dual use (cigarettes and e-cigarettes)				
None or not a member	ref	ref	ref	ref
Less than an hour	2.34	0.090	0.88	6.25
1-3 hrs	3.40	0.014	1.29	8.99
4-6 hrs	4.52	0.003	1.65	12.37
7 or more	5.31	0.002	1.84	15.33

AOR = adjusted odds ratio, CI = confidence interval

Results from models controlled for year, age, sex, country in UK, self-defined ethnic group (White vs. non-White), an indicator of living in an urban or rural areas, and Index of Multiple Deprivation in five groups. Cigarette smoking models were adjusted for cigarette smoking by caregivers and e-cigarette models by use of e-cigarettes by caregivers. Dual use models were adjusted for use of both e-cigarettes and cigarettes by caregivers.

Appendix Table 5: Associations of social media use with e-cigarette use from Generalised Estimating Equation models, categorising regular e-cigarette use as at least monthly use

Weekday social media use (hours/day)	AOR	Lower CI	Upper CI
Current e-cigarette use			
None or not a member	ref	ref	ref
Less than an hour	1.70	0.020	1.09
1-3 hours	2.16	0.001	1.35
4-6 hours	3.45	<0.001	2.14
7 or more	3.65	<0.001	2.18

Results from model controlled for year, age, sex, country in UK, self-defined ethnic group (White vs. non-White), an indicator of living in an urban or rural areas, equivalised household net income and use of e-cigarettes by caregivers.

AOR = adjusted odds ratio, CI = confidence interval

Appendix Table 6: Associations of social media use with e-cigarette use from Generalised Estimating Equation models, controlling for GHQ as a measure of mental health

Weekday social media use (hours/day)	AOR	p value	Lower CI	Upper CI
Current cigarette smoking				
None or not a member	ref	ref	ref	ref
Less than an hour	1.72	0.028	1.06	2.79
1-3 hrs	2.01	0.004	1.24	3.25
4-6 hrs	2.02	0.005	1.23	3.29
7 or more	2.57	<0.001	1.56	4.23
GHQ*	1.05	<0.001	1.04	1.06
Current e-cigarette use				
None or not a member	ref	ref	ref	ref
Less than an hour	0.60	0.286	0.24	1.53
1-3 hrs	0.76	0.572	0.30	1.94
4-6 hrs	1.00	0.996	0.39	2.56
7 or more	0.93	0.876	0.36	2.42
GHQ*	1.06	0.002	1.02	1.10
Current dual use (cigarettes and e-cigarettes)				
None or not a member	ref	ref	ref	ref
Less than an hour	0.87	0.832	0.24	3.15
1-3 hrs	1.18	0.792	0.34	4.15
4-6 hrs	1.08	0.910	0.30	3.90
7 or more	1.45	0.577	0.39	5.34
GHQ*	1.13	<0.001	1.08	1.19

***** per one unit increase in General Health Questionnaire-12, a widely used measure of population mental health. Greater scores indicate greater mental distress

Results from models controlled for , GHQ-12 scores, year, age, sex, country in UK, self-defined ethnic group (White vs. non-White), an indicator of living in an urban or rural areas, and equivalised household net income. Cigarette smoking models were adjusted for cigarette smoking by caregivers and e-cigarette models by use of e-cigarettes by caregivers. Dual use models were adjusted for use of both e-cigarettes and cigarettes by caregivers.

Appendix Table 7: Fixed effect models of changes in social media use and uptake of cigarette smoking and e-cigarettes

Note these from two separate models controlled for changes in household income (continuous) and in cigarette/e-cigarette use by parents. In cigarette model N = 864, in e-cigarette model N = 564.

Current cigarette smoking				
	AOR	p value	Lower CI	Upper CI
Weekday social media use (hours/day)	ref	ref	ref	ref
less than an hour	1.69	0.068	0.96	2.99
1-3 hrs	2.10	0.010	1.21	3.66
4-6 hrs	2.01	0.019	1.13	3.56
7 or more	2.33	0.006	1.28	4.24
Current e-cigarette use				
Weekday social media use (hours/day)	ref	ref	ref	ref
less than an hour	0.71	0.363	0.34	1.48
1-3 hrs	0.81	0.565	0.40	1.66
4-6 hrs	1.02	0.949	0.49	2.15
7 or more	0.84	0.669	0.38	1.85

AOR = adjusted odds ratio, CI = confidence interval
